# Characterization of a P-Rex1 gene signature in breast cancer cells

**DOI:** 10.18632/oncotarget.10285

**Published:** 2016-06-24

**Authors:** Laura Barrio-Real, Eva Wertheimer, Rachana Garg, Martin C. Abba, Marcelo G. Kazanietz

**Affiliations:** ^1^ Department of Systems Pharmacology and Translational Therapeutics, Perelman School of Medicine, University of Pennsylvania, Philadelphia, USA; ^2^ Centro de Estudios Farmacológicos y Botánicos (CEFYBO), Universidad de Buenos Aires, Buenos Aires, Argentina; ^3^ Centro de Investigaciones Inmunológicas Básicas y Aplicadas (CINIBA), Facultad de Ciencias Médicas, Universidad Nacional de La Plata, La Plata, Argentina

**Keywords:** P-Rex1, Rac1, heregulin, MMP10, breast cancer

## Abstract

The Rac nucleotide Exchange Factor (Rac-GEF) P-Rex1 is highly expressed in breast cancer, specifically in the luminal subtype, and is an essential mediator of actin cytoskeleton reorganization and cell migratory responses induced by stimulation of ErbB and other tyrosine-kinase receptors. Heregulin (HRG), a growth factor highly expressed in mammary tumors, causes the activation of P-Rex1 and Rac1 in breast cancer cells via ErbB3, leading to a motile response. Since there is limited information about P-Rex1 downstream effectors, we carried out a microarray analysis to identify genes regulated by this Rac-GEF after stimulation of ErbB3 with HRG. In T-47D breast cancer cells, HRG treatment caused major changes in gene expression, including genes associated with motility, adhesion, invasiveness and metastasis. Silencing P-Rex1 expression from T-47D cells using RNAi altered the induction and repression of a subset of HRG-regulated genes, among them genes associated with extracellular matrix organization, migration, and chemotaxis. HRG induction of MMP10 (matrix metalloproteinase 10) was found to be highly sensitive both to P-Rex1 depletion and inhibition of Rac1 function by the GTPase Activating Protein (GAP) β2-chimaerin, suggesting the dependence of the P-Rex1/Rac1 pathway for the induction of genes critical for breast cancer invasiveness. Notably, there is a significant association in the expression of P-Rex1 and MMP10 in human luminal breast cancer, and their co-expression is indicative of poor prognosis.

## INTRODUCTION

Rho GTPases represent a key family within the superfamily of Ras-related small G-proteins that plays fundamental roles in biology and disease. Among the members of this family, Rac GTPases (Rac1, 2, and 3) have been widely implicated in numerous cellular functions, including the regulation of actin cytoskeleton dynamics, cell motility, and the progression through the cell cycle. Rac GTPases cycle between a GDP-bound inactive state and a GTP-bound active state that is responsible for activation of effectors. Rac activity is tightly regulated by guanine nucleotide exchange factors (Rac-GEFs), which activate Rac by promoting the exchange of GDP by GTP, and GTPase-activating proteins (GAPs) that accelerate Rac intrinsic GTPase activity, leading to its inactivation [1–3]. Dysregulation of the Rac pathway is a common event in human cancer and has been largely associated with metastasis in a number of cancer types. Although gain-of-function mutations in Rac and a constitutively active splice variant (Rac 1b) have been found in a number of cancer types [4–7], hyperactivation of Rac is for the most part associated with aberrant overexpression or hyperactivation of Rac-GEFs [8–11]. Functionally, Rac hyperactive status leads to enhanced activation of downstream effectors such as Pak1 and the consequent elevated migratory and invasive capacity of cancer cells, ultimately favoring metastatic dissemination [11–14].

In a previous study we identified P-Rex1 as a key Rac-GEF implicated in actin cytoskeleton reorganization and motility of breast cancer cells [15]. P-Rex1, which was originally discovered in neutrophils, is dually activated by the PI3K product PIP3 and Gbγ subunits upon stimulation of tyrosine-kinase and G-protein-coupled receptors [16–18]. Silencing the expression of P-Rex1 from breast cancer cells largely impairs the elevation in Rac-GTP levels in response to growth factors such as EGF (ErbB1/EGFR ligand) and heregulin/neuregulin-1 (HRG, ErbB3/ErbB4 ligand), drastically affecting the motile capacity of these cells [15, 19]. Interestingly, P-Rex1 is highly overexpressed in breast cancer cell lines, specifically in those of luminal origin. P-Rex1 overexpression only occurs in a subset of human breast cancers, namely luminal A and B subtypes, which may be the consequence of altered epigenetic regulatory mechanisms that result in demethylation of the *PREX1* gene promoter [20]. P-Rex1 up-regulation in breast cancer has been linked with a high probability of metastatic dissemination in patients [15].

Dysregulation of the expression of ErbB receptors and their ligands is a common feature in many human cancers. Hyperactivation of the pathways controlled by ErbB receptors leads to uncontrolled growth, transformation, and enhanced motility and invasion. EGFR mutations are indeed major drivers of cancer progression, and ErbB2 amplification is a common oncogenic event in breast cancer [21–23]. ErbB3 also plays important roles in cancer, as it dimerizes preferentially with ErbB2 to confer an oncogenic signal [22, 24]. It has been reported that overexpression of HRG, which occurs in a significant fraction of mammary tumors, contributes to the tumorigenic and invasive capacities of breast cancer cells, even in the absence of ErbB2 overexpression [25, 26]. In support of the oncogenic role of HRG in breast cancer, transgenic overexpression of this growth factor in mouse mammary glands leads to the development of adenocarcinomas [27]. Notably, HRG regulates the expression of immediate early genes in estrogen receptor positive cells, including genes regulated by MAPK and the PI3K pathways, both established effectors of ErbB receptors [28]. The ErbB3 pathway has been implicated in the resistance to anticancer agents and is among the kinome pathways reprogrammed during drug resistance [22, 29, 30], thus highlighting the crucial need for dissecting downstream effectors of this receptor network.

Given the relevance of the P-Rex1/Rac1 pathway in breast cancer progression, and since the Rac pathway controls a number of key functions implicated in gene expression [31–33], we decided to pursue an analysis of genes regulated by P-Rex1 in breast cancer cells. As stimulation of ErbB3 by HRG causes a prominent activation of Rac1 in luminal breast cancer cells via P-Rex1, leading to a motile response, we investigated how silencing P-Rex1 could affect the regulation of gene expression by this growth factor. Our results identified a characteristic profile of P-Rex1-regulated genes in breast cancer cells, arguing for the involvement of the P-Rex1/Rac1 pathway in the control of gene expression and breast cancer progression.

## RESULTS

### Gene expression changes induced by HRG in T-47D breast cancer cells

**Figure 1:**
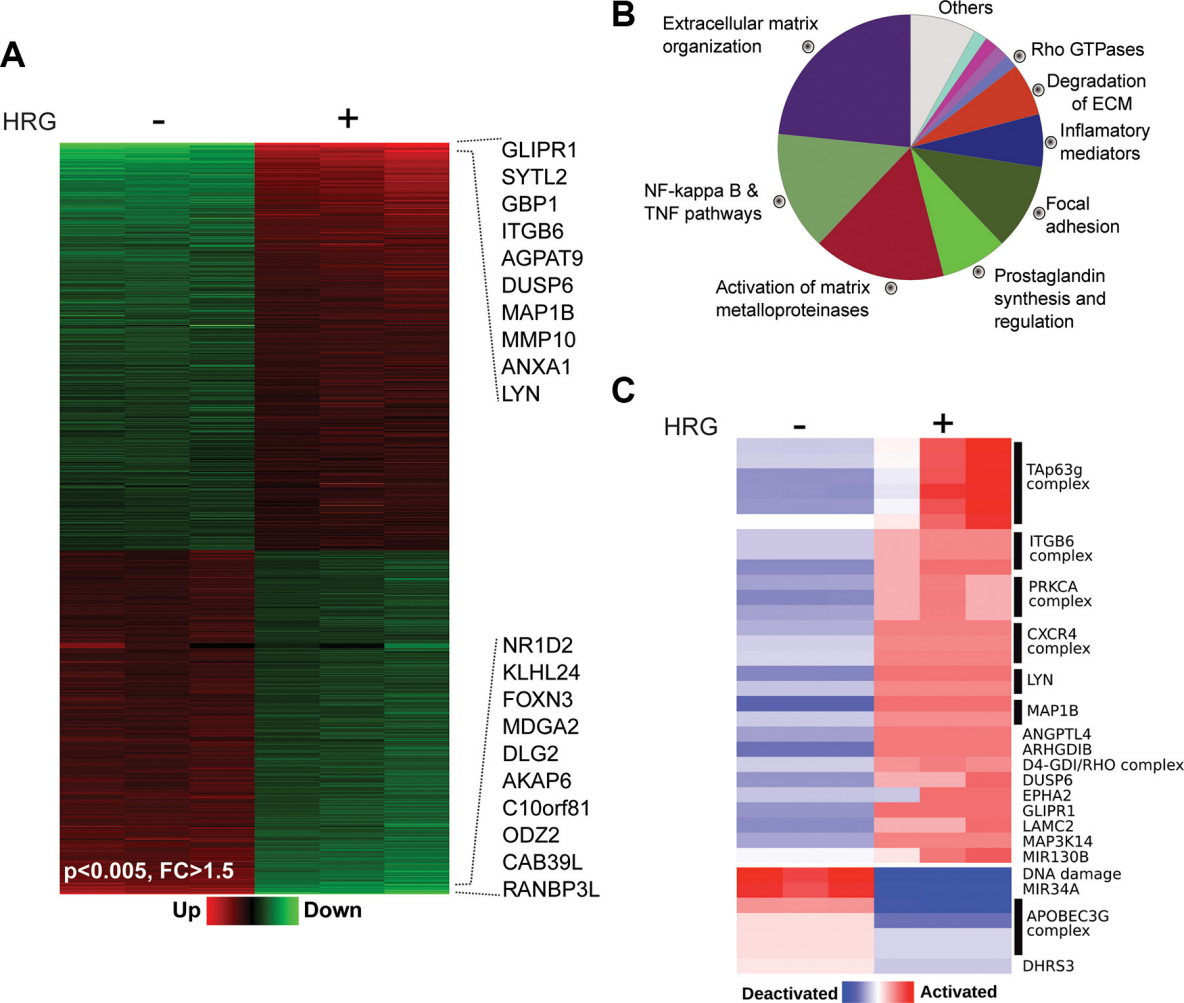
Changes in gene expression induced by HRG in T-47D cells T-47D cells were treated with HRG (20 ng/ml) or vehicle for 6 h. Total RNA from three replicates was extracted and reverse transcribed to cDNA. Gene expression profiling was carried out using an Affymetrix GeneChip Human Gene 1.0 ST Array. (**A**) Heatmap of the microarray data showing changes in gene expression by HRG (*p*-values < 0.005, fold changes (*FC*) > 1.5). The top 10 up- and down-regulated genes are indicated. (**B**) Enriched biofunctions of HRG-modulated genes, as determined by ClueGO analysis. The percentage of genes per term is proportionally represented in the pie chart. (**C**) PARADIGM inferences of the most variable integrated pathway activities using the normalized gene expression profiles of the HRG modulated transcripts.

As a first step in our search for mechanisms by which P-Rex1 mediates HRG responses in breast cancer, we first set to explore global changes in gene expression induced by this growth factor. Towards this end, we carried out a microarray analysis of genes regulated by HRG in T-47D breast cancer cells. Cells were treated with HRG (20 ng/ml) or vehicle, and 6 h later RNA from three replicates was extracted and reverse transcribed to cDNA. Gene expression profiling was carried out using an Affymetrix GeneChip Human Gene 1.0 ST Array (which includes more than 28,000 genes). Using a 1.5-fold change relative to vehicle-treated cells as a cut-off, we found that 1130 genes (represented by 1176 probes) were differentially expressed after HRG treatment relative to vehicle (*p*-values < 0.005, fold-change (FC) > 1.5). Among the transcripts modulated by HRG in T-47D cells, 516 were up-regulated and 614 were down-regulated (Figure 1A). A complete list of HRG-regulated genes is presented in Supplementary Table S1. The top 20 up- and down-regulated genes are shown in Table 1. The highest inductions were for *GLIPR1* (~30-fold), a gene hormonally-regulated in breast cancer cells [34], and *SYTL2* (~12-fold), a trafficking protein also known as Breast Cancer-Associated antigen SGA-72M [35]. Several top up-regulated genes were associated with motility, adhesion, invasiveness, and metastasis in breast cancer or other cancers, such as *GBP1*, *ITGB6*, *ITGA2*, and matrix metalloproteases *MMP10* and *MMP1*.

**Table 1:** Genes regulated by HRG in T-47D cells

Gene Name	Entrez ID	Description	Fold change
**Top twenty HRG up-modulated genes**			
*GLIPR1*	11010	*GLI pathogenesis-related 1*	30.8
*SYTL2*	54843	*Synaptotagmin like 2*	12.6
*GBP1*	2633	*Guanylate binding protein 1, interferon-inducible*	8.3
*ITGB6*	3694	*Integrin subunit beta 6*	6.5
*AGPAT9 (GPAT3)*	84803	*1-acylglycerol-3-phosphate O-acyltransferase 9*	5.7
*DUSP6*	1848	*Dual specificity phosphatase 6*	5.7
*MAP1B*	4131	*Microtubule associated protein 1B*	5.4
*MMP10*	4319	*Matrix metallopeptidase 10*	5.3
*ANXA1*	301	*Annexin A1*	4.7
*LYN*	4067	*LYN proto-oncogene, Src family tyrosine kinase*	4.6
*MMP1*	4312	*Matrix metallopeptidase 1*	4.5
*SERPINA3*	12	*Serpin peptidase inhibitor, clade A member 3*	4.5
*MIR21*	406991	*MicroRNA 21*	4.4
*KRTAP3-1*	83896	*Keratin associated protein 3-1*	4.3
*SPRR1A*	6698	*Small proline-rich protein 1A*	4.1
*SLC16A9*	2200963	*Solute carrier family 16 member 9*	4.1
*EPGN*	255324	*Epithelial mitogen*	3.9
*ITAG2*	3673	*Integrin subunit alpha 2*	3.9
*IL6R*	3570	*Interleukin 6 receptor*	3.9
*SERPINB8*	5271	*Serpin peptidase inhibitor, clade B member 8*	3.8
**Top twenty HRG down-modulated genes**			
*RANBP3L*	202151	*RAN binding protein 3-like*	−6.5
*CAB39L*	81617	*Calcium binding protein 39 like*	−5.1
*ODZ2 (TENM2)*	57451	*Teneurin transmembrane protein 2*	−4.1
*C10orf81 (PLEKHS1)*	79949	*Pleckstrin homology domain containing S1*	−3.5
*AKAP6*	9472	*A-kinase anchoring protein 6*	−3.5
*DLG2*	1740	*Discs, large homolog 2*	−3.5
*MDGA2*	161357	*MAM domain glycosylphosphatidylinositol anchor 2*	−3.3
*FOXN3*	1112	*Forkhead box N3*	−3.2
*KLHL24*	54800	*Kelch like family member 24*	−3.2
*NR1D2*	9975	*Nuclear receptor subfamily 1 group D member 2*	−3.1
*CDH10*	1008	*Cadherin 10, type 2 (T2-cadherin)*	−3.0
*ANK3*	288	*Ankyrin 3, node of Ranvier (ankyrin G)*	−2.9
*LRRC31*	79782	*Leucine rich repeat containing 31*	−2.9
*BLNK*	29760	*B-cell linker*	−2.9
*VEPH1*	79674	*Ventricular zone expressed PH domain containing 1*	−2.8
*TMEM229B*	161145	*Transmembrane protein 229B*	−2.8
*ACOT6*	641372	*Acyl-CoA thioesterase 6*	−2.7
*GRIK4*	2900	*Glutamate receptor, ionotropic, kainate 4*	−2.6
*ANKFN1*	162282	*Ankyrin repeat, fibronectin III domain containing 1*	−2.6
*CREB3L4*	148327	*cAMP responsive element binding protein 3-like 4*	−2.5

The table depicts the top 20 up- and down-regulated genes by HRG in T-47D cells, with the corresponding changes in expression.

Functional enrichment analysis for the categorization of HRG regulated genes identified “Extracellular matrix (ECM) organization/degradation”, “Activation of matrix metalloproteinases” and “Regulation of NF-kappa B and TNF pathways” as the predominant biofunctions associated with gene expression changes (Figure 1B). This is consistent with the well-established association of ErbB receptors, including ErbB3, with metastatic processes as well as with cytokine/pro-inflammatory pathways that play important roles in breast cancer progression [22, 36, 37]. In addition, we carried out a pathway-based representation analysis (PARADIGM) of deregulated transcripts, which led to the identification of signaling pathways that were specifically activated or inactivated by HRG (Figure 1C). One of the pathways identified with this analysis is the CXCR4 pathway. Notably, we reported that HRG-induced activation of P-Rex1/Rac1 and motility via ErbB3 in breast cancer cells is mediated by transactivation of CXCR4 [15], a G-protein-coupled receptor widely involved in metastatic dissemination [38]. The PARADIGM analysis also revealed the activation of PRKCA (PKCα), ITGB6 (integrin β6), LYN, and TAp63g pathways. The APOBEC3G complex involved in DNA deamination was among the pathways deactivated by HRG.

### P-Rex1 controls gene expression in breast cancer cells

**Figure 2:**
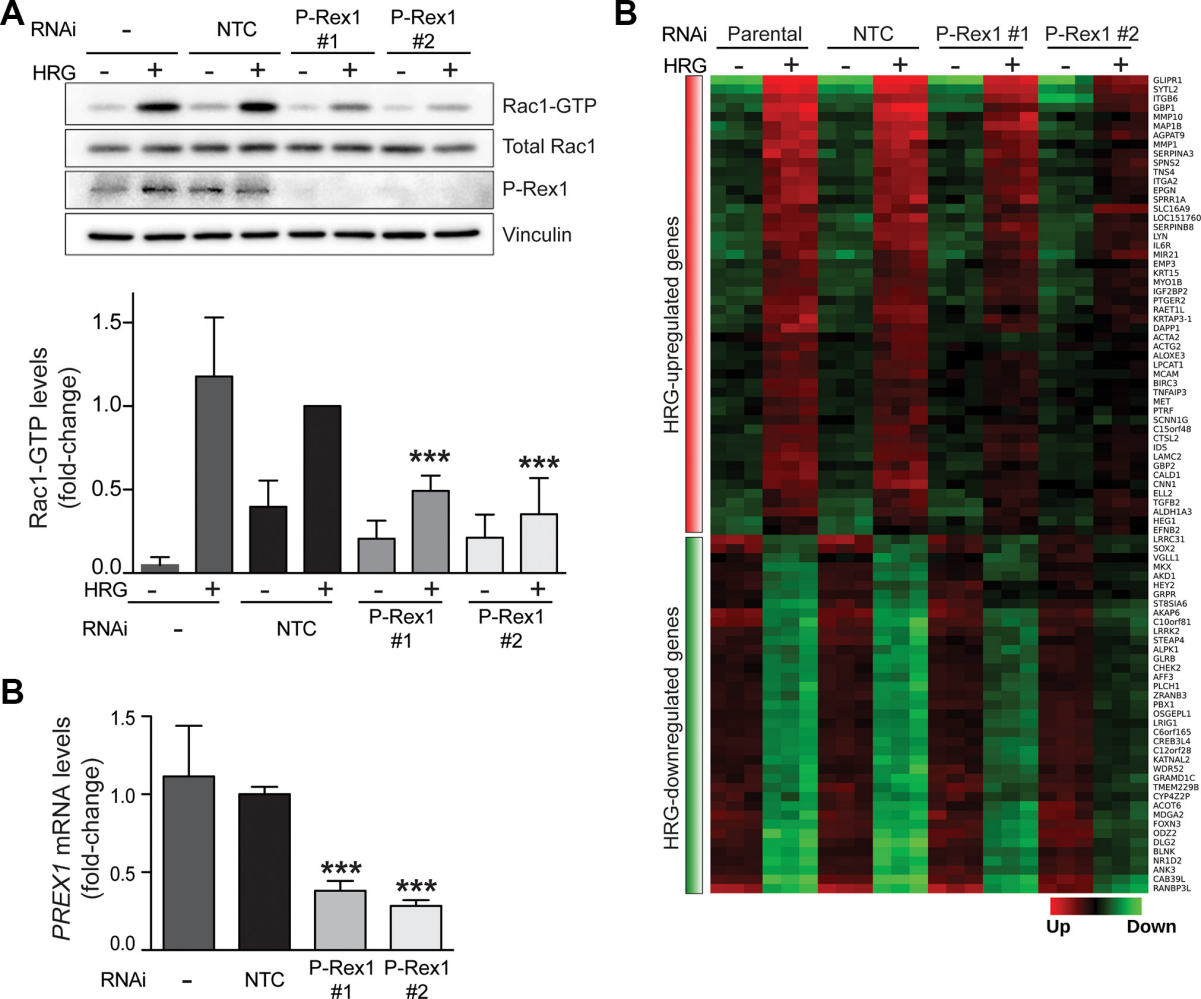
Depletion of P-Rex1 from T-47D cells using RNAi T-47D cells were transfected with two different P-Rex1 RNAi duplexes (*P-Rex #1* and *P-Rex #2*) or a non-target control RNAi duplex (*NTC*). After 16 h, cells were serum starved for 48 h and stimulated with HRG (20 ng/ml) or vehicle for 5 min. (**A**) Rac1-GTP levels in response to HRG were determined using a pull-down assay. *Upper panel*, representative experiment. *Lower panel*, densitometric analysis of Rac1-GTP levels normalized to total Rac1. Data (mean ± S.E.M., *n* = 3) are expressed as fold-change relative to NTC, +HRG. ***p < 0.005. (**B**) Determination of *PREX1* mRNA levels by qPCR. Expression was normalized to the housekeeping gene *B2M*. Data was expressed as fold-change relative to NTC. The experiment was performed in triplicate samples. Similar results were obtained in two additional independent experiments. ***p < 0.005. (**C**) Heatmap of genes regulated by HRG that are sensitive to P-Rex1 RNAi depletion. (*q* < 0.001; FC >1.5).

In previous studies we demonstrated that the Rac-GEF P-Rex1 is overexpressed in luminal breast cancer cells. We showed that HRG causes significant activation of P-Rex1 in breast cancer cells through activation of ErbB3 [15, 39]. Among the multiple Rac-GEFs expressed in luminal breast cancer cells, P-Rex1 was found to be key for the activation of Rac1 as well as for driving cell motility, growth and tumorigenesis downstream of ErbB receptors [15]. To assess the potential contribution of P-Rex1 in gene expression mediated by HRG, we next carried out a microarray analysis in T-47D cells subject to P-Rex1 RNAi depletion. As most luminal breast cancer cells, T-47D cells express high P-Rex1 levels [15]. To silence P-Rex1 expression, we used two different RNAi duplexes (P-Rex1 #1 and P-Rex1 #2). A non-target RNAi duplex (NTC) was used as control. There was a significant reduction in P-Rex1 protein and mRNA levels upon transfection with either P-Rex1 RNAi duplex, as determined by Western blot and qPCR analysis, respectively. On the other hand, no significant changes in P-Rex1 expression could be observed in cells transfected with NTC RNAi relative to parental T-47D cells (Figure 2A and 2B). In agreement with previous studies [15], P-Rex1 RNAi depletion caused a marked reduction in HRG-induced activation of Rac1, as determined by measuring Rac1-GTP levels using a pull-down assay (Figure 2B).

**Figure 3:**
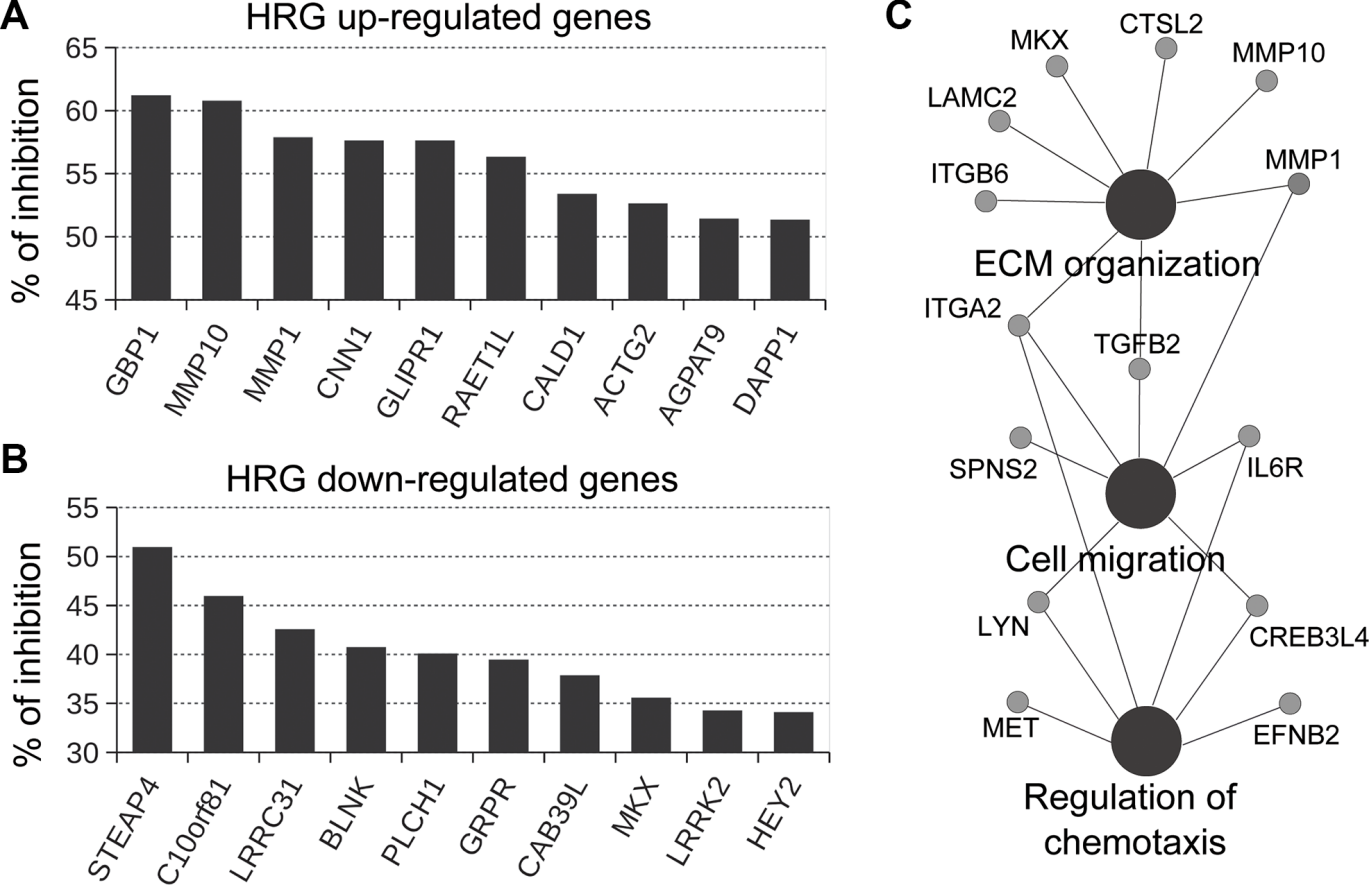
Effect of P-Rex1 RNAi on the expression of genes regulated by HRG T-47D cells were transfected with two different P-Rex1 RNAi sequences (*P-Rex #1* and *P-Rex #2*), or a non-target control RNAi (*NTC*). After 16 h, cells were serum starved for 48 h and stimulated with HRG (20 ng/ml) or vehicle for 6 h. Gene expression profiling was carried out using an Affymetrix GeneChip Human Gene 1.0 ST Array. P-Rex1-regulated genes were defined as those in which both P-Rex1 RNAi duplexes (#1 and #2) caused a statistically significant change (*p* < 0.05) in gene expression compared to NTC and parental cells. (**A**) Effect of P-Rex1 RNAi on genes induced by HRG. (**B**) Effect of P-Rex1 RNAi on genes repressed by HRG. For A and B, results are expressed as % of inhibition by P-Rex1 RNAi of the induction (for A) or repression (for B) in gene expression caused by HRG. Only the top 10 P-Rex1 regulated genes are shown. (**C**) CluePedia network of functionally enriched pathways and genes modulated by the HRG/P-Rex1 pathway in T-47D cells.

P-Rex1-regulated genes were defined as those in which both P-Rex1 RNAi duplexes caused a statistically significant change (*p* < 0.05) in gene expression compared to NTC and parental cells. This analysis revealed a set of 89 HRG-regulated genes that were sensitive to P-Rex1 RNAi, as displayed in the heatmap shown in Figure 2C. Among these genes, 50 were positively regulated by P-Rex1 (i.e., induction by HRG was reduced upon P-Rex1 silencing) and 39 were negatively regulated by P-Rex1 (i.e., repression by HRG was reduced upon P-Rex1 silencing). A complete list of P-Rex1-regulated genes is presented in Supplementary Table S2. The top 10 genes affected by P-Rex1 RNAi are ranked in Figure 3A and 3B. Several genes with the largest induction by HRG were markedly affected as a consequence of P-Rex1 silencing, among them the matrix metalloproteases *MMP10* and *MMP1*, *GBP1*, and *GLIPR1*. We next carried out a functional enrichment analysis to identify biofunctions associated with P-Rex1 regulated genes. This analysis revealed “Extracellular matrix organization”, “Cell migration” and “Regulation of chemotaxis” as the most significant functions linked to P-Rex1 (Figure 3C).

### MMP10 expression is regulated by the P-Rex1/Rac1 pathway

**Figure 4:**
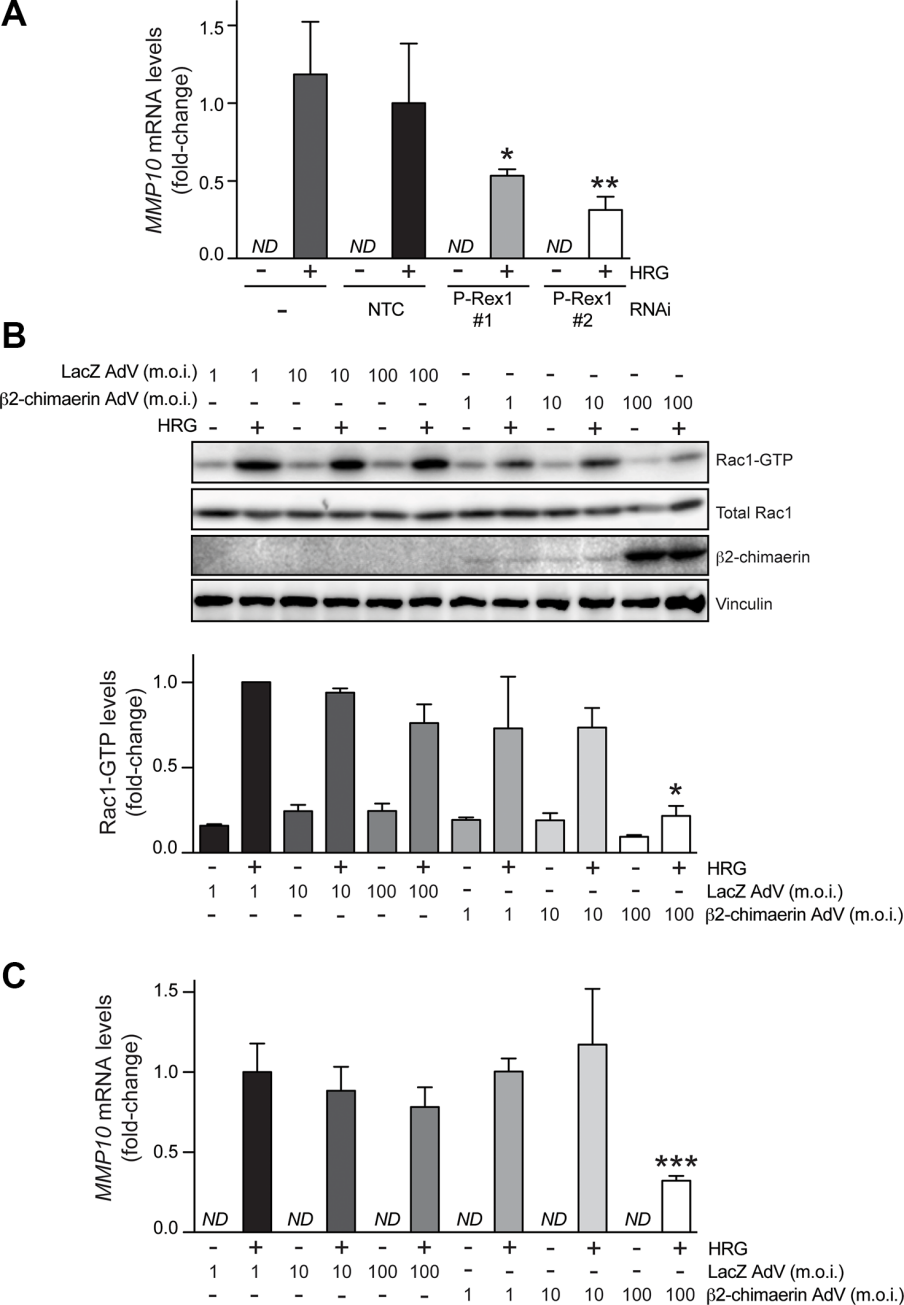
Induction of *MMP10* by HRG is mediated by P-Rex1 (**A**) T-47D cells were transfected with two different P-Rex1 RNAi duplexes (*P-Rex #1* and *P-Rex #2*), or a non-target control RNAi duplex (*NTC*). After 16 h, cells were serum starved for 48 h and stimulated with HRG (20 ng/ml) or vehicle for 6 h. *MMP10* mRNA levels were determined by qPCR. Expression was normalized to the housekeeping gene *B2M*. Data was expressed as fold-change relative to NTC, +HRG. The experiment was performed in triplicate samples. Similar results were obtained in two additional independent experiments. *p < 0.05; **p < 0.01 *vs.* NTC, +HRG. *ND*, non detectable. (**B**) Effect of β2-chimaerin on HRG-induced activation of Rac1. T-47D cells were serum starved for 48 h and then infected with AdVs for either β2-chimaerin or LacZ (control) using different m.o.i.'s (1–100 pfu/cell). After 16 h, cells were stimulated with either HRG (20 ng/ml) or vehicle for 5 min, and Rac1-GTP levels were determined using a pull-down assay. *Upper panel*, representative experiment. *Lower panel*, densitometric analysis of Rac1-GTP levels normalized to total Rac1. Data (mean ± S.E.M., *n* = 3) are expressed as fold-change relative to cells infected with LacZ AdV (m.o.i. = 1 pfu/cell), + HRG (*lower panel*). *p < 0.05 *vs.* LacZ AdV 100 m.o.i., +HRG. (**C**) *MMP10* mRNA levels were determined by qPCR in response to HRG (20 ng/ml) or vehicle treatment (6 h). Data was normalized to the housekeeping gene *B2M*, and expressed as fold-change relative to cells infected with LacZ AdV (m.o.i. = 1 pfu/cell), +HRG. The experiment was performed in triplicate samples. Similar results were obtained in two additional independent experiments. ***p < 0.005 *vs.* LacZ AdV 100 m.o.i. + HRG. *ND*, non detectable.

In order to validate results from the microarray analysis, we chose MMP10, which has been previously implicated in metastasis in breast cancer cells as well as in other cancer types [40–44]. A qPCR approach was developed to assess MMP10 mRNA levels. We observed that MMP10 expression could not be detected in T-47D cells under basal conditions. Upon HRG treatment, a significant elevation in MMP10 expression levels was observed. Consistent with data from the microarray analysis, the induction of MMP10 by HRG was significantly reduced in T-47D cells subject to P-Rex1 RNAi depletion (Figure 4A). Similar results were observed in SK-BR3 breast cancer cells, which express high P-Rex1 levels (Supplementary Figure S1).

Next, we asked if the effects of P-Rex1 on gene expression are mediated by Rac. We reasoned that impairing the GDP/GTP nucleotide exchange should affect MMP10 induction by HRG. To test this hypothesis, we ectopically expressed β2-chimaerin, a Rac-specific GAP that accelerates GTP hydrolysis from Rac1 leading to its inactivation, as extensively demonstrated in cell-free systems and cell lines, including T-47D breast cancer cells [14, 45]. We infected T-47D cells with the β2-chimaerin adenovirus (AdV) at different multiplicities of infection (m.o.i. = 1–100 pfu/cell). As a control, we used a LacZ AdV [45]. Significant overexpression of β2-chimaerin was achieved, particularly at m.o.i. = 100 pfu/cell (Figure 4B). As expected, and in agreement with previous reports [14, 45], expression of β2-chimaerin inhibited Rac1 activation by HRG, as judged by the reduction in Rac1-GTP levels. At m.o.i. = 100 pfu/cell, which impaired Rac activation by 65 %, the β2-chimaerin AdV significantly inhibited MMP10 induction (60% reduction compared with LacZ AdV at the same m.o.i.) (Figure 4C). Therefore, consistent with the requirement of P-Rex1 for MMP10 induction, these results indicate that in breast cancer cells MMP10 expression is regulated by Rac1 activation. MMP10 could also be induced upon activation of the G-protein-coupled receptor CXCR4 with SDF-1, which also leads to Rac1 activation (Supplementary Figure S2).

### Correlation of MMP10 and P-Rex1 expression in human luminal breast cancer

**Figure 5:**
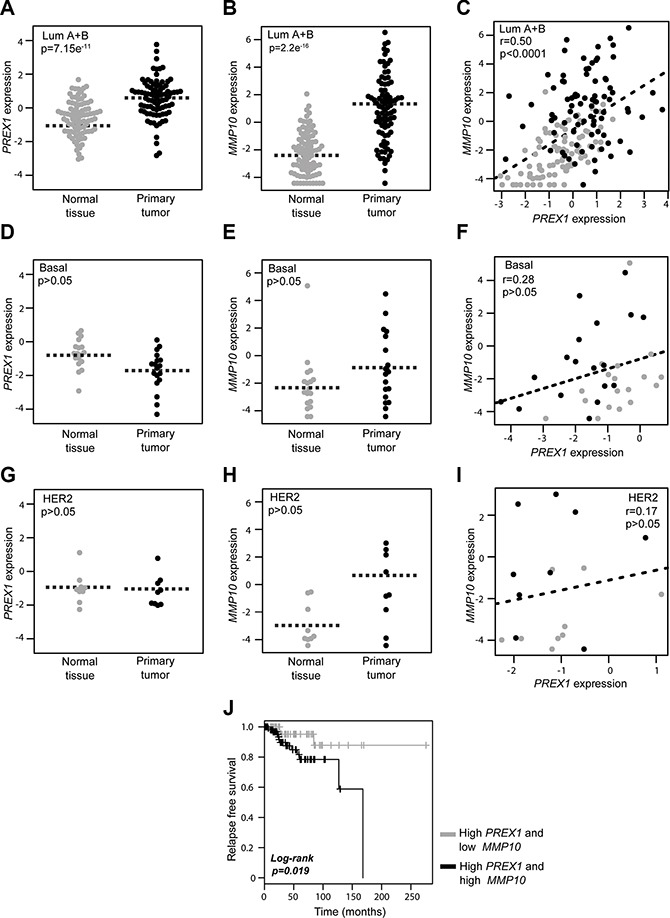
Expression of *PREX1* and *MMP10* in different breast cancer subtypes Comparison of *PREX1* and *MMP10* expression in paired breast carcinomas and normal tissues according to subtype was carried out using the TCGA breast cancer data obtained from the Xena resource. (**A**, **D**, and **G**) Expression of *PREX1* in luminal, basal, and HER2 positive breast cancer. (**B**, **E**, and **H**) Expression of *MMP10* in luminal, basal, and HER2 positive breast cancer. (**C**, **F**, and **I**) Correlation between *PREX1* and *MMP10* expression in luminal, basal, and HER2 positive breast cancer. (**J**) Kaplan-Meier curve analysis for P-Rex1 positive cases according to low and high *MMP10* expression.

In order to determine if potential associations between P-Rex1 and MMP10 exist in human breast cancer, we first performed a comparative analysis of *PREX1* and *MMP10* expression in paired breast carcinomas and normal tissue, using the TCGA (The Cancer Genome Atlas) breast cancer data obtained from the UCSC Xena resource (http://xena.ucsc.edu/). Consistent with our previous immunohistochemistry and bioinformatics analyses [15, 20], RNA-seq expression data revealed higher *PREX1* levels in luminal breast cancer specimens relative to normal samples from the same patients (*p* = 7.15e–11, *n* = 168) (Figure 5A). Similarly, *MMP10* levels are elevated in luminal breast tumors compared to their normal counterparts (*p* = 2.2e–16) (Figure 5B). Most notably, as shown in Figure 5C, there was a significant positive correlation between *PREX1* and *MMP10* levels in luminal breast cancer (*r* = 0.50; *p* < 0.0001). On the other hand, a similar analysis carried out in basal breast cancer specimens revealed that *PREX1* levels were not significantly different (*p* > 0.05, *n* = 36) between cancer and normal tissue (Figure 5D), which is consistent with our previous study [15, 20]. Likewise, expression of *MMP10* did not differ (*p* > 0.05) between basal cancer and normal samples (Figure 5E). As shown in Figure 5F, there was no correlation between *PREX1* and *MMP10* in basal breast cancer (*r* = 0.28; *p* > 0.05). Despite the low number of samples from HER2 positive patients available for this analysis, there were no obvious differences in the expression of *PREX1* (Figure 5G) and *MMP10* (Figure 5H) in this breast cancer subtype (*p* > 0.05, *n* = 18), nor a significant correlation between *PREX1* and *MMP10* expression could be found (*r* = 0.17; *p* > 0.05) (Figure 5I).

Lastly, to add prognostic value to our studies, we asked if *MMP10* expression in patients could be linked to survival. Luminal A breast cancer patients with high *PREX1* levels from which follow-up data was available were divided into two groups, low *MMP10* and high *MMP10* levels, based on the median expression values. As shown in Figure 5J, Kaplan-Meier analysis revealed that the subgroup of patients with high *MMP10* expression was associated with shorter recurrence-free survival compared to those with low expression of the *MMP10* gene (*p* = 0.019). These data suggest that expression of *MMP10* in luminal tumors may greatly influence patient outcome, and that high *MMP10* levels are an indicator of poor long-term survival.

## DISCUSSION

Studies from our group and others reported that P-Rex1, a Rac-GEF originally discovered in neutrophils [16], is highly expressed in a subset of breast cancers, specifically in the luminal subtype, and that its expression is associated with the development of breast cancer metastasis [15, 19]. The link with metastasis is supported by the presence of P-Rex1 positive cancer cells in lymph nodes of breast cancer patients [15]. More recent studies highlighted important roles for P-Rex1 in invasiveness of prostate cancer and melanoma cells [46, 47]. Functional studies revealed that P-Rex1 plays essential roles in the control of actin cytoskeleton reorganization and breast cancer cell motility driven by activation of ErbB receptors. In this context, the relevance of ErbB tyrosine-kinase receptors in breast cancer is well established. Indeed, ErbB2 amplification is a hallmark of human breast cancer, and a number of ErbB ligands are highly expressed in breast tumors, including the EGFR ligand TGFα and the ErbB3/ErbB4 ligand HRG [21, 22, 24, 48, 49]. Both ErbB2 overexpression and ErbB3 activation by HRG have been linked to breast cancer metastasis [25]. The main goal of this study was to identify genes regulated by P-Rex1 in the context of HRG stimulation in breast cancer cells, with the ultimate goal of establishing effector pathways implicated in P-Rex1 function.

In this study, we first focused on the profiling analysis of genes regulated by HRG in breast cancer cells. As anticipated from the multiplicity of pathways activated by ErbB ligands, HRG stimulation of T-47D breast cancer cells led to marked changes in gene expression, both leading to up- and down-regulation. It is not surprising that HRG-regulated genes include many associated with extracellular matrix reorganization/degradation and cytokine function, which is consistent with the well-established roles of HRG in conferring an invasive and pro-metastatic phenotype. Pathway-based representation analysis using PARADIGM revealed the activation of pathways known to be associated with ErbB receptor function and breast cancer progression, such as ITGB6. The ITGB6 gene, coding for the integrin subunit β6, has been associated with metastasis to distant sites and poor prognosis in breast cancer patients, and targeting avβ6 integrin has been proposed as a novel therapeutic approach for treating high-risk and trastuzumab-resistant breast cancer patients [50]. A second interesting finding relates to the identification of the PRKCA (PKCα) pathway as a HRG effector. PKCα has been functionally associated with ErbB2, the main dimerization partner for ErbB3, and was found to be an essential mediator of ErbB2-driven breast cancer invasiveness [51, 52]. The PARADIGM analysis also identified the CXCR4 pathway as activated by HRG. In our previous study [15], we established that HRG-induced activation of P-Rex1/Rac1 and motility in luminal breast cancer cells is mediated by transactivation of CXCR4, a G-protein-coupled receptor widely associated with breast cancer cell metastatic dissemination. Indeed, HRG treatment promotes the phosphorylation and activation of CXCR4, leading to the release of Gbγ subunits from heterotrimeric Gi proteins that are required, in conjunction with PI3K stimulation, to activate P-Rex1/Rac1 and confer a motile response [17]. It is important to note that a previous microarray analysis by Amin *et al.* [28] characterized HRG-regulated genes in T-47D cells, although this study focused primarily on immediate early genes that in most cases do not overlap with our analysis. The differences may relate to the fact that our study involved a longer HRG treatment (6 h), as we aimed to recapitulate a chronic growth factor stimulation scenario that occurs in human breast tumors.

The identification of P-Rex1 regulated genes in breast cancer is also a notable finding. We identified a subset of 89 HRG-modulated genes sensitive to P-Rex1 RNAi depletion. Although functional studies to characterize the role of these specific genes in the HRG/P-Rex1 pathway are beyond the scope of the current study, the biofunctions associated with P-Rex1, which we identified through functional enrichment analysis, emphasize the relevance of the pathway in cell migration, regulation of chemotaxis, and extracellular matrix organization. Previous studies in neutrophils established that P-Rex1 serves as a regulator of chemotaxis in response to a variety of extracellular stimuli [53]. In addition, studies in various cancer cell lines, including breast cancer cells, established key roles for P-Rex1 in cell migration stimulated by growth factors such as HRG as well as GPCR ligands such as SDF-1, a chemokine that activates CXCR4 [15, 19, 54]. Silencing P-Rex1 from breast cancer cells impairs HRG-induced cytoskeleton reorganization and ruffle formation, processes that are key for cellular motility [15]. Emerging evidence in a number of cancer types indicates that P-Rex1 is involved in metastatic dissemination. The first evidence was found using prostate cancer cell xenografts, where expression of P-Rex1, but not a GEF dead P-Rex1 mutant, induced lymph node metastasis [46]. In addition, P-Rex1 knockout mice are resistant to metastasis when crossed to a murine model of melanoma [47]. Expression of P-Rex1 also promotes an invasive behavior in human fibroblasts [55]. The involvement of P-Rex1 in breast cancer invasiveness is still poorly understood, probably due to the limited availability of invasive luminal cellular models; nevertheless P-Rex1 expression has been associated with a higher probability of developing metastasis, and P-Rex1 positive cells can be readily detected in lymph nodes of breast cancer patients [15]. In this context, our microarray analysis identified a number of gene products associated with the remodeling of the extracellular matrix, among them MMP10. Although MMP10 has been less studied compared to other MMPs, its expression has been shown in human specimens of luminal A breast tumors [56]. Moreover, a role for MMP10 has been proposed in MCF-7 breast cancer cells via regulation of the BAD protein [40]. Interestingly, a recent study established that MMP10 contributes to hepatocarcinogenesis driven by CXCR4 stimulation [41]. Our results showing that SDF-1 activate Rac1 and induce MMP10 in breast cancer cells are in line with this finding. A role for MMP10 downstream of ErbB2 has been also demonstrated in pancreatic adenocarcinoma [42]. In addition, MMP10 has been implicated in lung cancer initiation driven by oncogenic K-Ras [44, 57]. Although the detailed mechanisms by which P-Rex1/Rac1 controls MMP10 expression are not known, we speculate that the effect may be mediated by the ERK pathway. Indeed, ERK has been shown to be a downstream effector of P-Rex1 in breast cancer cells and mediates MMP10 induction in other cell types [41, 58, 59]. Future studies in our laboratory are aimed at dissecting potential roles for MMP10 and other relevant genes identified in our analysis in breast cancer progression. It is also interesting that our Kaplan-Meier analysis revealed a shorter recurrence-free survival in high *PREX1* patients with high *MMP10* expression relative to those with low *MMP10* expression, indicating that *MMP10* expression can greatly influence patient outcome. The significant association between *PREX1* and *MMP10* expression in luminal tumors, together with the poor prognosis found in luminal breast cancer patients with high *PREX1* and *MMP10* expression, suggest a causal relationship with disease progression that deserves to be fully explored.

In summary, we identified the P-Rex1/Rac1 pathway as a regulator of the expression of genes in luminal breast cancer. One attractive possibility is that actin cytoskeleton reorganization, which is tightly regulated by P-Rex1/Rac1, contributes to the observed expression in genes. Indeed, there is extensive evidence that gene expression is tightly controlled by actin cytoskeleton dynamics [60]. Our studies emphasize the complexities of Rac-GEF effector mechanisms in cancer and suggest that phenotypes driven by this pathway may involve the regulation of genes implicated in various aspects of cell motility, invasiveness and extracellular matrix remodeling.

## MATERIALS AND METHODS

### Cell culture and reagents

T-47D and SK-BR3 cells were obtained from ATCC (Manassas, VA) and were grown in DMEM medium (Lonza; Basel, Switzerland) supplemented with 10% FBS (Hyclone; Logan, UT). Heregulin β1 and SDF-1 were obtained from R&D (Minneapolis, MN).

### RNAi transfection and adenoviral infection

For transient depletion of P-Rex1, we used ON-TARGETplus siRNAs J-010063-11 (#1) and J-010063-12 (#2), or siRNA pool L-010063-01-0005 (Dharmacon; Lafayette, CO). ON-TARGETplus non-targeting siRNA (D-001810-01) or non-targeting siRNA pool (D-001810-10) were used as controls (NTC). RNAi duplexes were transfected into T-47D cells using Lipofectamine RNAiMAX (Invitrogen; Carlsbad, CA) following the instructions provided by the manufacturer. After 16 h, cells were serum starved, and 48 h later subject to HRG treatment.

For adenoviral infections, T-47D cells were serum starved for 32 h, and then infected with different multiplicities of infections (m.o.i.) of either β2-chimaerin or LacZ (control) AdV, as in previous studies [45, 61]. Experiments were carried out 16 h after adenoviral infection.

### Rac1-GTP pull-down and Western blot assays

After serum starvation for 48 h, cells were stimulated with HRG (20 ng/ml) or SDF-1 (100 ng/ml) for 5 min. Rac1-GTP levels were determined with a pull-down assay using the p21-binding domain (PBD) of Pak1, as described previously [62]. Briefly, cells were lysed in a buffer containing 20 mM Tris-HCl, pH 7.4, 150μM NaCl, 5 mM MgCl_2_, 0.5% NP40, 5 mM β-glycerophosphate, 1 mM DTT, protease inhibitors, and 10 mg/ml GST-PBD. Lysates were cleared by centrifugation (10 min at 4°C, 13,000 × g) and incubated with glutathione-Sepharose 4B beads (GE Healthcare, Mickleton, NJ) for 45 min at 4°C. After centrifugation, the beads were washed twice with the pull-down buffer and run on SDS-PAGE gels.

For Western blot analysis, the following antibodies were used: anti-Rac1 (Upstate Biotechnology; Lake Placid, NY), anti-P-Rex1 and anti-vinculin (Sigma-Aldrich; St. Louis, MO). Bands were visualized by Enhanced Chemiluminescence (ECL). Images were captured using an Odyssey Fc system (Li-Cor Biosciences; Lincoln, NE). Image processing and densitometry analysis were carried out using the Image Studio Lite software (Li-Cor Biosciences).

### Microarray data processing, statistical and data mining analysis

For gene expression analysis, T-47D cells were serum starved and then stimulated with HRG (20 ng/ml) or vehicle (PBS) for 6 h in 60-mm plates. Three biological replicates for each experimental condition were used. For the analysis of P-Rex1-regulated genes, parental, NTC, P-Rex1#1 and P-Rex1#2 T-47D cells, with and without HRG treatment, were used (24 samples). Total RNA for each sample was obtained using the miRNeasy kit (Qiagen; Valencia, CA), reversed transcribed to cDNA (GeneChip^®^ Whole Transcript cDNA Synthesis and Amplification Kit, Affymetrix; Santa Clara, CA), fragmented, and end labeled (GeneChip^®^ WT Terminal Labelling Kit, Affymetrix) for further hybridization to an Affymetrix GeneChip Human Gene 1.0 ST Array (GPL6244) according to the standard protocols at the University of Pennsylvania Molecular Profiling Facility. RNA quality was analyzed using the Agilent Bioanalyzer Nano assay. We carried out QC and normalization procedures in R/Bioconductor using the “simpleaffy” package [63]. The Robust Multichip Average algorithm was employed for background adjustment, quantile normalization and probe set values summarization [64].

To compare T-47D parental (no RNAi) and NTC RNAi groups vs. P-Rex1 RNAi #1 and P-Rex1 RNAi #2, with and without HRG treatment, we utilized the Rank Products test [65]. Statistical analysis and heatmap visualization of differentially expressed transcripts were done with the MultiExperiment Viewer software (MeV 4.9) [66]. The complete microarray data are available in the Gene Expression Omnibus database (GSE77974). We used ClueGO and CluePedia Cytoscape's plug-in for functional enrichment analysis, network generation and visualization of the gene expression changes [67]. In addition, InnateDB resource (http://www.innatedb.com/) was employed for over representation analysis of GO terms based on the list of deregulated genes.

Pathway-based analysis was performed using the PARADIGM software [68] at the Five3 Genomics server (default options; discretization bounds of 33%) on the basis of the normalized gene expression profiles of the deregulated transcripts associated with HRG treatment in T-47D parental cells. PARADIGM produces a data matrix of integrated pathway activities (IPA). This data matrix was used in place of the mRNA expression profiles to identify the topmost variable IPAs among groups.

### Real-time quantitative PCR (qPCR)

Total RNA from cells was isolated using the RNeasy Mini Kit (Qiagen). One μg of total RNA was reverse transcribed to cDNA with the TaqMan Reverse Transcription Kit (Invitrogen). qPCR was performed in a ABI PRISM 7700 detection system using TaqMan Universal PCR MasterMix (Applied Biosystems, Branchburg, NJ), target primers (45 nM), fluorescent probe (12.5 nM) and the cDNA. TaqMan probes specific for *PREX1*, *MMP10* and the housekeeping gene *B2M* (used for normalization) were purchased from Applied Biosystems. PCR product formation was continuously monitored using the Sequence Detection System software version 1.7. Results were expressed as fold-change of the target gene by 2^−ΔΔCt^ and normalized to the NTC sample. All qPCR reactions were performed in triplicate. Every experiment was performed three times.

### *In silico* analysis of *PREX1* and *MMP10* expression in breast carcinomas

Comparative analysis of *PREX1* and *MMP10* mRNA expression in human breast samples was done in paired breast carcinomas and normal tissue, using the TCGA breast cancer data obtained from the UCSC Xena resource (http://xena.ucsc.edu/). *PREX1* and *MMP10* RNA-seq expression profiles were compared in normal and tumor sample using a paired *t*-test. Correlation analysis between both genes among normal and breast cancer samples was done with a Pearson's test.

Prognostic value of *PREX1* and *MMP10* profiles was evaluated in a subset of breast cancer patients with follow-up data. Patients with primary invasive breast carcinomas were divided into two groups (high *PREX1*/low *MMP10* and high *PREX1*/high *MMP10*) based on the median gene expression values. These groups were then compared for recurrence free survival using the Survival R package.

### Additional statistical analysis

Statistical analysis of qPCR data (−ΔΔC_t_ values) and densitometry data from Rac1-GTP pull-down assays were done by ANOVA and Bonferroni's multiple comparison tests, using the GraphPad Prism software.

## SUPPLEMENTARY MATERIALS FIGURES AND TABLES






