# Supplementary material for: Characterization of a P-Rex1 gene signature in breast cancer cells
Source: Oncotarget. 2016 Jun 24;7(32):51335–48. doi: 10.18632/oncotarget.10285 (PMC5239479; doi:10.18632/oncotarget.10285)
Supplement: Supplementary file 1 [file oncotarget-07-51335-s001.pdf]

Characterization of a P-Rex1 gene signature in breast cancer cells

Supplementary Materials

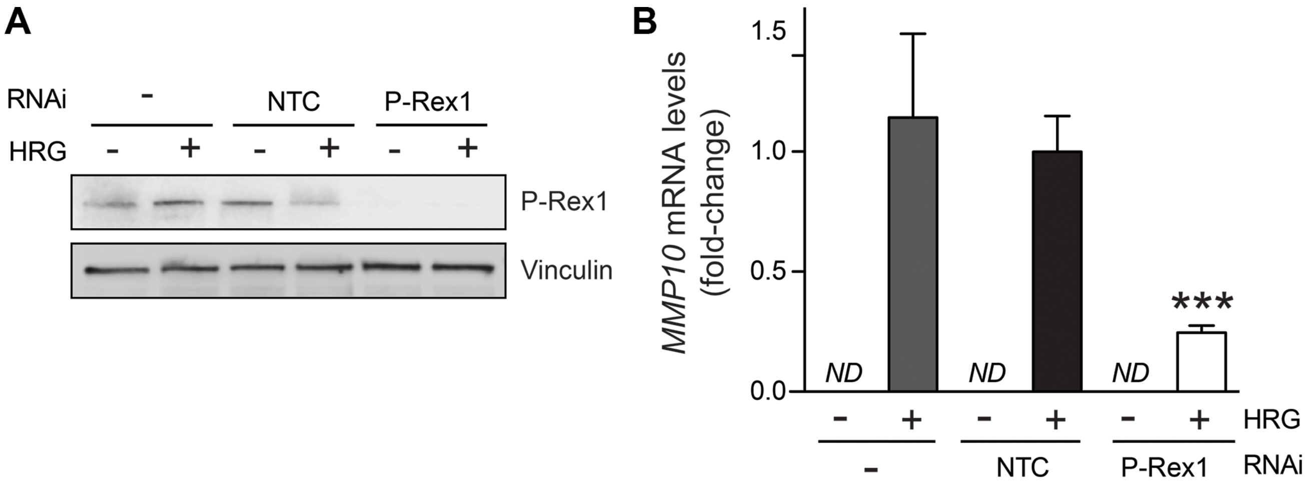

Supplementary Figure S1: Effect of P-Rex1 RNAi on *MMP10* induction in SK-BR3 cells. SK-BR3 cells were transfected with a P-Rex1 RNAi pool (Dharmacon) or a non-target control RNAi pool (*NTC*). After 16 h, cells were serum starved for 24 h and stimulated with HRG (20 ng/ml) or vehicle for 6 h. (**A**) P-Rex1 depletion as determined by Western blot. (**B**) Determination of *MMP10* mRNA levels by qPCR. Expression was normalized to the housekeeping gene *B2M*. Data was expressed as fold-change relative to NTC. The experiment was performed in triplicate samples. Similar results were obtained in two additional independent experiments. ***$p < 0.005$.

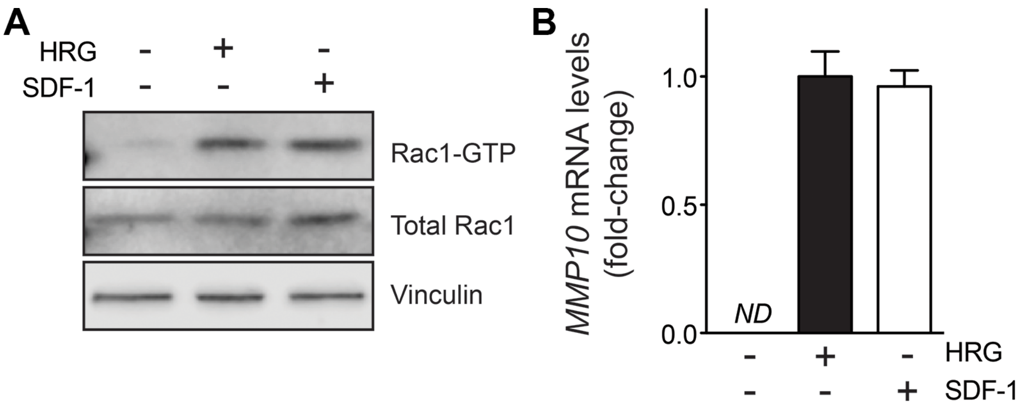

Supplementary Figure S2: SDF-1 induces *MMP10* in T-47D cells. (**A**) T-47D cells were serum starved for 24 h and stimulated with HRG (20 ng/ml), SDF-1 (100 ng/ml) or vehicle for 5 min. Rac1-GTP levels were determined using a pull-down assay. Similar results were obtained in two additional independent experiments. (**B**) Determination of *MMP10* mRNA levels by qPCR after 6 h stimulation with HRG, SDF-1 or vehicle. Expression was normalized to the housekeeping gene *B2M*. Data was expressed as fold-change relative to the HRG response. The experiment was performed in triplicate samples. Similar results were obtained in two additional independent experiments. *ND*, non detectable.

Supplementary Table S1: List of genes regulated by HRG in T-47D cells. The corresponding changes in gene expression are shown (*p*-values < 0.005, FC > 0.5). See Supplementary_Table_S1

Supplementary Table S2: List of genes regulated by P-Rex1 in T-47D cells. P-Rex1-regulated genes were defined as those in which both P-Rex1 RNAi duplexes (#1 and #2) caused a statistically significant change ($p < 0.05$) in gene expression compared to NTC. The percentage of inhibition relative to the induction/repression caused by HRG treatment is shown. See Supplementary_Table_S2
